# Urea dilution of serum for reproducible anti-HSV1 IgG avidity index

**DOI:** 10.1186/s12879-019-3769-x

**Published:** 2019-02-14

**Authors:** Jan Olsson, Jörgen Johansson, Emma Honkala, Bert Blomqvist, Eloise Kok, Bodil Weidung, Hugo Lövheim, Fredrik Elgh

**Affiliations:** 1 Department of Clinical Microbiology, Virology, Umeå University, SE-901 85 Umeå, Sweden; 2 Laboratory Medicine, Clinical Microbiology, Umeå University Hospital, Umeå, Sweden; 3 Tampere University Hospital, Tampere, Finland; 4 Department of Public Health and Caring Sciences, Geriatric Medicine, Uppsala University, Uppsala, Sweden; 5 Department of Community Medicine and Rehabilitation, Geriatric Medicine, Umeå University, Umeå, Sweden

**Keywords:** Herpes simplex, IgG, Avidity, ELISA, Primary infection, Reactivated infection

## Abstract

**Background:**

Herpes simplex virus type 1 (HSV1), establishes life-long latency and can cause symptoms during both first-time infection and later reactivation. The aim of the present study was to describe a protocol to generate a reliable and discriminative avidity index (AI) for anti-HSV1 IgG content in human sera.

**Methods:**

Human serum from two distinct cohorts; one a biobank collection (Betula) (*n* = 28), and one from a clinical diagnostics laboratory at Northern Sweden University Hospital (NUS) (*n* = 18), were assessed for presence of IgG antibodies against HSV1 by a commercially available ELISA-kit. Addition of urea at the incubation step reduces effective binding, and the ratio between urea treated sample and non-treated sample was used to express an avidity index (AI) for individual samples.

**Results:**

AI score ranged between 43.2 and 73.4% among anti-HSV1 positive biobank sera. Clinical samples ranged between 36.3 and 74.9%. Reproducibility expressed as an intraclass correlation coefficient (ICC) was estimated at 0.948 (95% CI: 0.900–0.979) and 0.989 (95% CI 0.969–0.996) in the biobank and clinical samples, respectively.

**Conclusion:**

The method allows for AI scoring of anti-HSV1 IgG from individual human sera with a single measurement. The least significant change between two measurements at the *p* < 0.05 level was estimated at 5.4 and 3.2 points, respectively, for the two assessed cohorts.

## Background

Herpes simplex is ubiquitously spread within the human population. Seroprevalence of anti-HSV1 IgG in adult populations is reported at approximately 80% [1–3]. Infection occurs through direct person-to-person contact, and susceptible persons may be infected anytime throughout life [3]. Several studies from Europe and the U.S.A. have reported a decreasing age-adjusted prevalence, indicative of a decreasing childhood and adolescent risk of HSV1 infection in the population [1, 3, 4]. This trend might be accompanied with a higher incidence of primary infections among adults, but this is not well studied. Neonatal transmission from mothers with genital HSV infection is a substantial risk, especially when the mother is infected for the first time close to birth [5]. The infection cycle typically involves a primary infection, often located at the mucosal surface of the oral cavity or the genital tract, followed by infection of sensory nerve endings and retrograde spread to neural ganglia where the virus establishes life-long latency. Reactivation may occur, resulting in viral shedding and sometimes extensive mucosal viral replication. This process is often subclinical, but may also cause symptoms such as cold sores. Antibody avidity, i.e. the compound value for binding strength for a pool of multivalent antigens and antibodies, normally increases during the maturation of an immune response. Avidity testing has diagnostic value, especially in identifying cases of genital HSV with risk for neonatal infections [5–7]. Recently, as HSV1 infection suggests an increase in the risk of developing Alzheimer’s disease [8–13], avidity testing might provide further predictive value [14–16]. ELISA kits, used to assess serum for presence of anti-HSV IgG, are provided by several manufacturers, including kits that distinguish anti-HSV1 from anti-HSV2. Through the addition of a washing step including a chaotropic salt, some HSV2-kits have been validated to also provide avidity data. HSV1 type specific kits have been experimentally modified in a similar manner by several research groups [5, 6, 14, 15], but no validated kit is, to the best of our knowledge, currently commercially available. For use in future studies, we established the current protocol, HSV1 avidity index (HSV1 AI) with the aim of reproducible determination of anti-HSV1 IgG avidity.

## Methods

ELISA kits for HSV1 IgG (Vir-Elisa Anti-HSV1-IgG, ref. 102) were obtained from VIRO-IMMUN, Oberursel, Germany. (Now: DIAsource ImmunoAssays S.A, Oberursel, Germany). We have performed a validation study where we found the sensitivity and specificity of this ELISA to be on par with the in house method [17], with antigen from HSV1-infected GMK cells, we use for routine analysis. Both methods show cross-reaction with anti-HSV2 IgG, as judged by a parallel run with the HSV1 & HSV2 type-specific HerpeSelect® IgG ELISA methods (Focus Diagnostics, California, USA) based on recombinant glycoprotein G. The sensitivity in scoring a serum positive or negative for presence of anti-HSV1 IgG was equal for these three methods [13].

Reagents were added manually with an 8-channel pipette and wells were washed with an automatic plate washer (Wellwash Versa Microplate Washer, ThermoScientific, Waltham, Massachusetts, USA). From a biobank collection (Betula) [18] of serum from adults (age 35–95), 28 random samples were tested for presence of anti-HSV1 IgG, according to the protocol provided by the manufacturer. In short, serum were diluted 1:101, where after 100 μl was added to each well and incubated at room-temperature for 30 min. Washing of wells were performed with 300 μl washing buffer 4 times, with 30 s soaking time. Peroxidase conjugate (100 μl) was added and incubated for 30 min at room-temperature, where after wells were washed with 300 μl washing buffer 4 times, with 30 s soaking time. TMB substrate (100 μl) was added and incubated, shielded from light, for 10 min at room temperature. Stop solution (100 μl) was added, and the absorbance was read at 450 nm (reference 620 nm) in a plate reader (model “Sunrise”, Tecan, Männedorf, Switzerland). The kit is provided with a cut-off control to which samples are related (positive: ODsample > 1.1x ODcut-off).

Sixteen samples were positive and subjected to avidity evaluation. Washing with 6 M Urea is earlier reported as a method for assessment of avidity for anti-HSV1 IgG [5, 6, 14, 15] and thus we modified the protocol to include such a washing after the serum incubation step. The Urea solution (150 μl) was allowed to soak the wells containing ag-bound anti-HSV1 IgG for 8 min, as reported earlier [6]. By dividing the values for Absorbance (Urea/no Urea) for doublets run with and without the Urea washing step, an Avidity Index (AI) with theoretical values between 0 and 100% is generated. AI for the 16 samples varied between 65 and 95%. However, when samples were reinvestigated, the reproducibility was poor (Intraclass correlation coefficient [ICC] < 0.8, data not shown). A number of variations to the protocol were performed; Urea concentration 2-8 M, incubation time 30s - 20 min, agitation of the plate while soaking, soaking at 4-37C, application of the urea washing step after an initial washing-buffer step, use of 0.75 M NaSCN. The resulting spread of avidity for the 16 samples varied between 42 and 100%. None of the protocol variants improved the reproducibility in a significant way (Intraclass correlation coefficient [ICC] < 0.8, data not shown).

This prompted us to instead challenge the initial binding of anti-HSV1 IgG to the antigen coated on the ELISA plate by dilution of sera with chaotropic salt (the dilution principle). For each sample we thus prepared additional samples diluted 1:101 in 2 M, 3 M, and 4 M Urea, respectively. Samples were applied to the standard ELISA protocol as described above. Spread of avidity for the samples varied between 69 and 88% (2 M), 51–82% (3 M), and 44–73% (4 M) (data not shown). Re-runs indicated good reproducibility, and the 4 M dilution protocol was chosen for further evaluation (see discussion). In the final method serum samples were clarified by centrifugation (1000 x g, 10 min) where after 10 μl was transferred to tubes containing: a) 1 ml sample diluent (provided in the kit), and b) 1 ml 4 M Urea (CAS Number 57–13-6, analysis grade, EMD Millipore) in sample diluent, prepared the same day. Diluted samples (100 μl), a & b for each serum, were immediately added to the ELISA plate wells and processed as described above, i.e. 30 min incubation followed by 4x washing etc. Background signal was subtracted from Absorbance (OD_450_) values, and an AI value was estimated for each serum sample (Abs_UREA_/Abs_Diluent_). Repeats were performed with fresh dilutions.

## Results

**Fig. 1:**
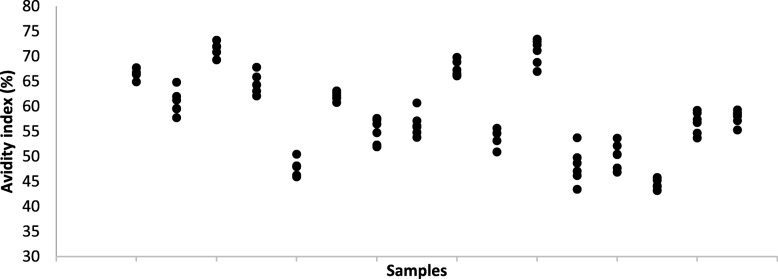
Biobank samples. Anti-HSV1 IgG avidity index for 16 biobank samples. Results from six independent runs

**Fig. 2:**
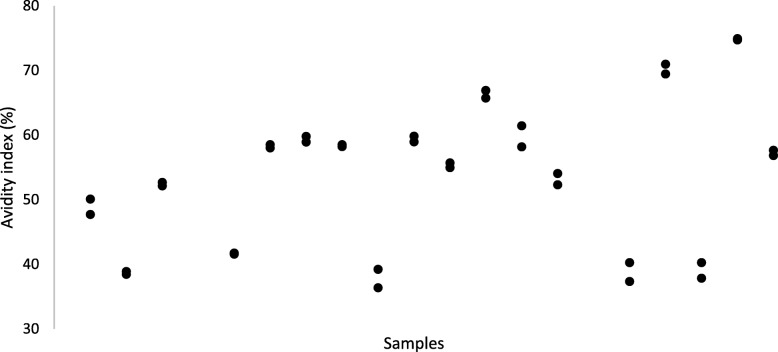
Clinical sera. Anti-HSV1 IgG avidity index for 18 patient sera. Assessment performed in duplicate

The 16 biobank samples showed AI values between 43.2 and 73.4%. Six independent repetitions showed good reproducibility (Fig. 1). To test the method on fresh serum, we followed up with 18 anti-HSV1 IgG positive de-identified patient sera from a clinical lab (Fig. 2). AI among these samples varied between 36.3 and 74.9%.

The ICC was calculated based on a single-rating, consistency, two-way random-effects model. ICC was estimated at 0.948 (95% confidence interval [CI] = 0.900–0.979) in the biobank sample and 0.989 (95% CI = 0.969–0.996) in the clinical sample, indicating very high relative reliability in both samples. Heteroscedasticity of the AI was tested using Pearson correlation between individual mean of AI and individual SD. The correlation was *r* = − 0.237 (*p* = 0.377) in the biobank sample and *r* = − 0.285 (*p* = 0.268) in the clinical sample, indicating homogenous SD across the AI in both samples. This was also investigated graphically by plotting individual mean of AI against individual SD, showing homogenous data. Within-subject standard deviation (s_w_), as a measure of absolute reliability was calculated by taking the square root of the residual mean square derived from an analysis of variance (ANOVA) table [19]. The s_w_ was 1.953 and the 95% CI for a single measurement ±3.827 in the biobank sample, and s_w_ 1.153 and 95% CI for a single measurement ±2.259 in the clinical sample. To assess repeatability of the AI, the least significant change between two measurements at the *p* < 0.05 level was calculated as 1.96s_w_ x √ 2, yielding biobank and clinical samples with the values: 5.412 and 3.195, respectively. All statistical analyses were performed using IBM SPSS Statistics software version 24 (IBM Corp. Armonk, NY).

## Discussion

Avidity assessment of the IgG population in human serum is a means for acquiring basic information on the quality of a humural response against e.g. a viral pathogen. The principle of avidity assessment in ELISA or immunofluorescence is straightforward, and includes a sequence in the protocol where binding of IgG to antigen is challenged with a chaotropic agent. Although the method is well established for numerous pathogens, we failed to find a commercially available ELISA kit validated for assessment of anti-HSV1 IgG avidity. Whether this reflects a low market demand or an inherent technical difficulty in producing such a kit, remains an open question. By a slight modification of a commercial whole-virion antigen ELISA-kit, we could achieve good segregation of individual sera with excellent reproducibility. Urea at a concentration of 4 M was chosen as IgG-antigen interfering agent because the range of AI for assessed sera then extended below 50%, a potentially useful breakpoint value for future dichotomous classification of sera as high- or low avid. It can by noted, though, that the AI range was marginally larger, 31 points vs 29 points, with 3 M urea. We had to turn to the lesser used dilution principle; elution steps with urea or other chaotropic salts did not perform nearly as well in our system. Dissociation of an already bound high-affinity IgG to its antigen requires concentrations of urea (6-8 M) that is denaturing the IgG molecule [20]. Albeit the denaturation is reversible, it is possible that the recognition of bound IgG by the HRP-conjugate is affected as a consequence of the denaturation taking place in an earlier step, and thus that random molecular events such as proper protein refolding influence the repeatability. The lower concentrations of urea used in dilution protocols are hypothesised to spare tertiary structures [21] and that may be an explanation for the better repeatability. The protocol allows for a statistically significant stratification of human sera with a single measurement, a crucial criterion for our intended use, that is assessment of biobank cohorts. Further studies will reveal the correlates of AI to parameters such as age of the patient, IgG titers, IgM status, primary infection/reactivation etc. Reproducibility was a major concern during our attempts to adapt protocols from earlier studies, for example we could never achieve the reported level of an average change in the avidity value for repeated measurements of 6 points reported by Brown et al. [5]. It must be emphasised though, that the types of ELISA plates differ among studies.

## Conclusions

In conclusion, the human serum anti-HSV1 IgG avidity assay described here, utilising an additional 4 M urea dilution step to a whole-cell antigen based ELISA, was found to be reliable and discriminative for both fresh clinical samples and in Biobank serum samples.

## Abbreviations

AI: Avidity index
CI: Confidence Interval
ELISA: Enzyme Linked Immuno-Sorbent Assay
HSV1/ 2: Herpes simplex virus type 1/ 2
ICC: Intraclass Correlation Coefficient
IgG/M: Immunoglobulin G/M
SD: Standard Deviation
